# The Medical, Surgical, and Hygienic Exhibition

**Published:** 1897-07-31

**Authors:** 


### The Medical, Surgical, and Hygienic Exhibition.

This interesting exhibition has been opened at the Queen's Hall, where all the latest appliances and preparations connected with medicine and surgery are well displayed. With the object of rendering the exhibition still more attractive, and affording entertainment to the visitors, excellent instrumental music is provided both afternoon and evening.

On entering the hall the first stall on the right is occupied by Vimbos (Limited), whose preparations of meat extract are manufactured solely in Scotland. Among the precautions taken by this firm to ensure the absolute purity of their product is that of sending samples daily to the Medical Officer of Health for Edinburgh. Vimbos is also shown in the convenient form of lozenges for cyclists and athletes, which are both palatable and portable.

Hard by Messrs. John Dewar and Co., of Perth, display samples of their well-known whisky. One of the special features of their exhibit is an anti-diabetic whisky, which combines valuable medicinal properties with the flavour for which their brand is justly celebrated.

Both medical men and nurses will find considerable attraction in the exhibits of Mr. W. K. Stacy, prominent among which is a new thermometer carrier for a nurse's chatelaine, combining a pincushion specially constructed to carry safety-pins, with a case from which the clinical thermometers can be readily withdrawn, and as quickly replaced. A closed wallet, now in general use at Guy's Hospital, also merits attention from nurses.

Messrs. Brooks and Co. exhibit a new solution of iodine discovered by Mr. R. J. Downes, which is rapidly absorbed, and if rubbed in lightly till dry will not stain the skin.

The Peat Industries Syndicate (Limited) show samples of their Béraudine surgical dressing, now in general use in several of the metropolitan hospitals, and also of natural antiseptic towelettes and accouchement sheets of the same material. Among the advantages claimed for these preparations are their deodorising properties, and their power of absorbing and distributing moisture.

At the stall of the Williams Typewriter Company Mr. Owen P. O'Connor shows an instrument made by this company, with special signs for prescriptions. The mechanism is simple, the instrument light, and the type neat and clear.

Mr. B. Kühn exhibits his well-known disinfectant and antiseptic chinosol in many forms, and also various tubes and cylinders of ethyl chloride and anestile, showing the latest improvements, which are of considerable interest.

The Berkefeld Filter Company make a prominent feature of their aseptic irrigator, an apparatus for supplying sterilised water of regulated temperature for use in surgical operations.

Close by Fleming's Oil and Chemical Company (Limited) exhibit, among other things, their Camphylene Cream, a disinfectant with a pleasant odour which does not stain floors or carpets.

Messrs. Hockin, Wilson, and Co, Messrs. John Weiss, and Messrs. Mayer and Metzler present an imposing array of surgical instruments and appliances, while Messrs. Stephen Smith and Co. display their Hall's Coca Wine on a most tastefully decorated stall.

The Liebig Extract of Meat Company give prominence to their new invalid food, Peptarnis, which is manufactured from specially selected oxen, and has already been taken up by the leading metropolitan hospitals and infirmaries.

The Sanitary Wood Wool Company are well to the fore with a good show of their hygienic towelettes, sheets, and accouchement outfits, for which the firm is justly celebrated.

One of the most interesting exhibits is that of Messrs. Idris (Limited), showing the apparatus and tests used by them to secure the absolute purity and excellence of their waters.

Messrs. Alexander Riddle and Co. have a most refreshing exhibit of Stower's Lime Juice Cordial and of their more recent production, Stower's Clarified Lemon Squash.

The Aylesbury Dairy Company exhibit, in addition to their well-known milk and cream, a new food for infants under the name of Muffler's Food, which is prepared from milk, wheat-flour, and eggs, and sterilised *in vacuo*.

Izal may be seen at Messrs. Newton, Chambers, and Co.'s stall in the various useful forms in which this excellent disinfectant is now made up, not the least interesting being an aqueous solution of their pure oil which they have succeeded in obtaining, and of which they anticipate great things when perfected.

Among Messrs. Bailey and Son's novelties is a crape bandage which possesses the elasticity and strength of india-rubber without its attendant disadvantages. Their new "Verilite" belt and "Verilite" truss are also worthy of attention.

The Liverpool Lint Company make a special feature of their splint padding, an antiseptic dressing composed of carbolised tow and absorbent cotton wool combed together and placed between layers of gauze, the advantages of which are apparent. They also show their transferable vests, which they especially recommend for medical men.

At Messrs. Callard and Co.'s stall their diabetic foods may be seen and tasted in many varieties—from brown bread to chocolate, from cathartic biscuits to shortbreads.

Messrs. Warrick Bros'. exhibit of jelloids is most interesting to medical men. The most recent additions to their list include jelloids containing thyroid gland powder and red bone marrow and other organic products.

Messrs. Maw, Son, and Thompson have two stalls, at one of which they exhibit various surgical instruments and appliances, and at the other their accouchement sets.

The Bovril Company (Limited), who have recently taken over the Liquor Carnis preparations, exhibit a variety of their excellent food specialities, including their Bovril stamnoids, which should prove of excellent service to athletes and others who are compelled to go for any length of time without their ordinary meals.

The useful is combined with the ornamental at Madame Mona's stand, who exhibits a prettily got-up cot, all the bedding and hangings of which are made of antiseptic materials, and are manufactured by Madame Mona and Co.

Messrs. J. F. Macfarlan and Co. have an interesting and varied exhibit, including, as it does, Forret's Milk Steriliser for sterilising milk in small quantities, their Listerian surgical dressing, iodoform gauze, and Holyrood table water.

Messrs. Oppenheimer, Son, and Co. not only exhibit their well-known palatinoids and bi-palatinoids, but have loaned an unique collection of medical, surgical, and pharmaceutical antiquities, which occupy a room to themselves. These were collected by Dr. Sambon for the Italian Government in order to illustrate "The History of Medicine," which he was desired to prepare for presentation to the next meeting of the International Medical Congress. Amongst the objects shown were "donarii," taken from the medical springs and shrines. These were of clay, bronze, and silver. The clay images frequently represent portions of the human body, rudely fashioned by potters that sat by the wayside, though some were artistically modelled. The improvements in surgical instruments were seen at a glance, although the advance is nothing like so great as might have been expected, whilst some instruments, specula for instance, showed signs of retrogression during the fourteenth and following centuries. The vases and jars for medicaments, as well as tear bottles, were also represented.

The exhibits of Messrs. Lawes' Chemical Company

(Limited) should prove of interest to those responsible for the cleansing and disinfection of hospital wards and lavatories.

The cooling and refreshing qualities of Messrs. Rose and Co.'s lime juice can be practically experienced at their stall.

Messrs. Down Brothers, on their really handsome stall, exhibit a comprehensive collection of surgical instruments. They show also an excellent portable operation table devised by Mr. Jordan Lloyd.

Messrs. Parke, Davis, and Co. display a large collection of their well-known drugs and medical specialities, chief amongst which we noticed their taka-diastase.

American Laboratory Products were adequately represented by the exhibits of Messrs. Armour and Co., who give special prominence to their extract of beef.

The Rebman Publishing Company and Messrs. John Bale and Son exhibit books on medicine and surgery, while the Scientific Press (Limited), in addition to such works, show also books dealing with hospitals and nursing.

Among the exhibits which should not be overlooked are those of Messrs. Ingram and Royle, of Messrs. Fitton and Son (Hovis), and of the Mellin Food Company (Limited), whose specialities are too well known to need detailed notice here.

The exhibition, which is well worth a visit, remains open till Friday evening, July 30th.

